# VA-ECMO Supported Aspiration Thrombectomy for High-Risk PE: A Single-Center Experience

**DOI:** 10.1016/j.jscai.2024.102436

**Published:** 2024-11-13

**Authors:** Usman A. Hasnie, Chris Price, Meenakshi Sridhar, Megan Nordberg, Stephen Clarkson, Samuel McElwee

**Affiliations:** a Division of Cardiovascular Disease, Heersink School of Medicine, University of Alabama at Birmingham, Birmingham, Alabama; b Department of Medicine, Heersink School of Medicine, University of Alabama at Birmingham, Birmingham, Alabama; c Division of Pulmonary, Allergy and Critical Care Medicine, Heersink School of Medicine, University of Alabama at Birmingham, Birmingham, Alabama

**Keywords:** mechanical circulatory support, pulmonary embolism, venoarterial extracorporeal membrane oxygenation

High-risk pulmonary embolus (PE), defined by the 2019 European Society of Cardiology guidelines as PE with hemodynamic instability, right ventricular dysfunction on imaging, and elevated cardiac biomarkers, remains a challenging clinical scenario with a high mortality rate.^1^^,^^2^ In the setting of acute high-risk PE, specifically with severe right ventricular failure and refractory cardiogenic shock, initiation of venoarterial extracorporeal membrane oxygenation (VA-ECMO) can provide rapid hemodynamic stabilization. This hemodynamic support can provide crucial time to serve as a bridge to starting therapeutic anticoagulation or more definitive therapeutic options, such as surgical embolectomy and catheter-based therapies. However, data remain limited regarding aspiration thrombectomy as well as its deployment and management of ECMO adjunctively. To this aim, we performed a retrospective, single-center study at a large tertiary medical center examining the use of aspiration thrombectomy with VA-ECMO in patients diagnosed with high-risk PE from 2015 to 2022.

Over the study period, 38 patients with high-risk PE were cannulated for peripheral VA-ECMO. The arterial sheath size for each case was operator-dependent (ranging from 15F to 21F). The mean age was 51 years old, and 57.9% of patients were female. On presentation, 65.7% patients were tachycardic with heart rate >110 beats per minute, 36.8% had hypotension with systolic blood pressure <100 mm Hg, and 34.2% had oxygen saturation <90%. Prior to their cannulation for ECMO, 42.1% of patients suffered cardiac arrest. In total, 18 (47.4%) patients underwent adjunct aspiration thrombectomy with the FlowTriever System (Inari Medical) within the same procedure after ECMO initiation, the majority of which occurred the day of their presentation for medical care. Patients who did not undergo aspiration thrombectomy were medically managed, the majority (70%) receiving anticoagulation.

As detailed in Table 1, those that underwent aspiration thrombectomy were older (55 years vs 47 years); however, the rates of prior cardiovascular disease, prior venous thromboembolism, hypertension, coronary artery disease, diabetes, and heart failure were similar between groups. Additionally, the clinical presentation of both groups were similar, including the highest lactic acid (4.9 vs 7.4, *P* = .16) and right ventricle to left ventricle ratio on computed tomography scan (1.6 vs 1.7, *P* = .73) during their hospitalization.

**Table 1:** Baseline demographics, clinical characteristics and outcomes of patients with high risk PTE on VA-ECMO who underwent aspiration thrombectomy vs those that did not undergo thrombectomy from 2018 to 2024.

Covariate	Aspiration thrombectomy (n = 18)	No aspiration thrombectomy (n = 20)	*P*
Age, y	55.2 ± 13.9	47.4 ± 18.1	0.14
Female sex	9 (50.0)	13 (65.0)	0.51
Body mass index, kg/m^2^	32.3 ± 8.9	32.0 ± 7.4	0.92
Active smoker	4 (22.2)	2 (10.0)	0.39
Active malignancy	1 (5.6)	2 (10.0)	1
Recent trauma	2 (11.1)	3 (15.0)	1
Recent surgery	3 (16.7)	6 (30.0)	0.45
Vascular injury requiring intervention	2 (11.1)	3 (15.0)	1
AKI requiring dialysis	0 (0)	3 (15.0)	0.23
Hypertension	10 (55.6)	7 (35.0)	0.33
Coronary artery disease	1 (5.6)	0 (0)	0.47
Congestive heart failure	0 (0)	1 (5.0)	1
Diabetes mellitus	5 (27.8)	4 (20.0)	0.71
Stroke	1 (5.6)	3 (15.0)	0.61
History of VTE	3 (16.7)	2 (10.0)	0.65
HR >110 bpm	13 (72.2)	12 (60.0)	0.51
SBP <100 mm Hg	7 (38.9)	7 (35.0)	1
RR >30 bpm	7 (38.9)	4 (20.0)	0.29
Temperature <36°C	2 (11.1)	7 (35.0)	0.13
Altered mental status	7 (38.9)	11 (55.0)	0.35
SpO_2_ <90%	6 (33.3)	7 (35.0)	1
Syncope	5 (27.8)	4 (20.0)	0.71
BARC scale	3 (16.7)	2 (10.0)	0.65
RV: LV ratio on CT	1.6 ± 0.4	1.7 ± 0.5	0.73
Highest lactic acid before cannulation for ECMO, mmol/L	4.9 ± 4.1	7.4 ± 6.5	0.16
Pulmonary Embolism Severity Index	128.0 ± 49.8	127.9 ± 48.6	0.99
Received thrombolytics	4 (22.2)	8 (40)	
Mortality	1 (6)	9 (45)	0.0089
ICU length of stay, d	9.8 ± 11.2	27.8 ± 27.9	0.0132
Hospital length of stay, d	15.7 ± 15.3	30.5 ± 27.0	0.0447
Survived to discharge	17 (94.4)	12 (60.0)	0.02
Survived run	18 (100)	16 (80.0)	0.11
Alive at 30 d	17 (94.4)	14 (70.0)	0.09
Alive at 90 d	17 (94.4)	13 (65.0)	0.045
Alive at 1 y	17 (94.4)	10 (50.0)	0.0037

Values are reported as mean ± SD or n (%).

AKI, acute kidney injury; BARC, Bleeding Academic Research Consortium; CT, computed tomography; ECMO, extracorporeal membrane oxygenation; HR, heart rate; ICU, intensive care unit; RR, respiratory rate; RV: LV, right ventricular to left ventricular; SBP, systolic blood pressure; SpO_2_, saturation of peripheral oxygen; VTE, venous thromboembolism.

Patients that underwent aspiration thrombectomy had lower mortality (5.6% vs 45%, *P* = .009), shorter intensive care unit length of stay (9.8 days vs 28 days, *P* = .013), and shorter overall length of stay (15.7 days vs 30.5 days, *P* = 0.045) than those that did not receive aspiration thrombectomy. Patients that underwent aspiration thrombectomy had higher survival at 30 days, 90 days, and 1 year (Table 1).

In this study, we demonstrate encouraging results regarding the use of aspiration thrombectomy in critically ill patients with high-risk PE. Patients that received VA-ECMO for high-risk PE at our institution had significantly better outcomes when they received aspiration thrombectomy. Although the decision to pursue aspiration thrombectomy was influenced by operator/center experience, anatomically favorable clot, and a patient’s tolerance of ECMO, these data suggest that supported aspiration thrombectomy provides a mortality benefit. Current guidelines remain vague with regard to the use of catheter-based thrombectomy, thus far predominantly highlighting its use in patients that are not ideal candidates for thrombolytics due to high bleeding risk.^3^ Often, high-risk patients are excluded from randomized clinical trials of aspiration thrombectomy^4^; however, recently, Silver et al^5^ investigated the use of large bore mechanical thrombectomy in high-risk PE. These data are encouraging and suggestive that there was a low rate of in-hospital mortality with aspiration thrombectomy. We have further expanded on these data by specifically looking at patients with high-risk PE that required cannulation with VA-ECMO.

Although the data were extremely favorable for aspiration thrombectomy utilization, it must be noted that there have been contemporary developments including PE response team consultation/multidisciplinary approach, patient selection, and overall clinical management. While further exploration is needed, our study contributes to the utilization of aspiration thrombectomy as a potential therapeutic option for high-risk patients. Though retrospective in nature, our study captures real-world management of PE at a large quaternary academic institution that employs a PE response team.

In conclusion, we found that patients with high-risk PE, stabilized on VA-ECMO and undergoing aspiration thrombectomy, had shorter intensive care unit duration, shorter overall length of stay, and higher survival rate up to 1 year compared to those that were treated without thrombectomy in our small, single-center experience. Future larger or randomized trials are needed to better characterize the ideal population for aspiration thrombectomy among patients with high-risk PE, especially in those requiring aggressive mechanical support with VA-ECMO. Future studies will also need to evaluate the multiple devices that are being used for aspiration thrombectomy in these patients.
